# The impact of the digital economy on enterprise innovation behavior: Based on CiteSpace knowledge graph analysis

**DOI:** 10.3389/fpsyg.2023.1031294

**Published:** 2023-01-23

**Authors:** Wenling Yu, Lipai Zhang, Chen Yang

**Affiliations:** ^1^Jiangsu Provincial Academy of Social Sciences, Nanjing, China; ^2^School of Business and Management, Shanghai International Studies University, Shanghai, China; ^3^School of Management, Xiamen University, Xiamen, China

**Keywords:** innovation, digital economy, knowledge mapping, CiteSpace, Web of Science

## Abstract

**Introduction:**

As a new trend, the digital economy will promote “digital industrialization” in the process of promoting “industrial digitalization.” It can accelerate technological innovation by adjusting managerial behavior and strategic decisions, promoting and protecting technological research and development, and providing technological infrastructure. While technological innovation, which generally occurs in enterprises, will promote a new round of upgrading, optimization, and even reshaping of the whole industry. The two are highly synergistic. Therefore, it is of great practical significance to study the impact of the digital economy era on enterprise innovation behavior.

**Methods:**

We analyzed the impact of the digital economy era on corporate innovation behavior in the Web of Science database from 2010 to 2020 through bibliometric and scientific knowledge mapping methods.

**Results and discussion:**

Our study found that: the research on enterprise innovation behavior in the digital economy era has formed eight research directions, such as expertise, human capital FSA, integration in global value chains, financial innovation, fintech, people preference shift, internet of everything, and consumer co-creation. In addition, the research hotspots on enterprise innovation behavior in the digital economy era range from enterprises’ perception of digital economy contact, to enterprises’ familiarity with digital technology and its application, to enterprises’ attempted evolution of digital transformation, reflecting the potential of both theoretical and practical characteristics. Finally, we present an outlook on the future cross-sectional development of research on enterprise innovative behavior in the digital economy era and propose a research trend based on the Chinese context.

## 1. Introduction

Under the wave of economic globalization, production technology elements are surging worldwide. Only countries or regions with independent innovation ability can gain a foothold and sustainable development in the new round of technological revolution. At present, developing countries, including China, still face problems of low technology density and insufficient differentiation in their industrial cooperation in global value chain cooperation.

To adapt to the changes in global productivity and change the unfavorable position in the “smile curve,” since the 19th National Congress of the Communist Party of China, the Central Committee of the Communist Party of China has elevated innovation as “the first driving force to lead the development and the strategic support to build a modern economic system.” –Implementing the innovation-driven strategy, building an innovative country, strengthening the innovation system, and strengthening intellectual property rights have become important development strategies at the national level, and achieved initial results. According to the Global Innovation Index (GII) Report 2020 released by the World Intellectual Property Organization (WIPO), China maintains 14th place in the list of global innovation index among 131 economies in the world, the same as last year. In the same year, 17 technology clusters in China entered the top 100 global technology clusters, ranking 2nd after the United States. As the construction of socialism with Chinese characteristics deepens, enterprises in strategic transition, as micro-individuals in economic construction, their innovation capacity is also the driving force of long-term growth of the socialist economy. According to the new economic growth theory, innovation expenditures such as R&D investment are related to maintaining competitive advantages and forming comparative advantages (North and Knight, 1997). Under the current market economic system, enterprise innovation activities have gradually become an important indicator of their wealth creation and sustainable development (Berman et al., 2021; Zhou et al., 2021).

Corporate innovation activities have inherently economic uncertainty, most of which are capital expenditure (manifested as expenses in the short term), and the economic cycle is long. Executives take risks with such projects (Ding and Chea, 2021; Schneider, 2021). Especially conservative executives with a high degree of risk aversion need more internal and external incentives to compensate for losses and thus strengthen their willingness and motivation to participate in activities such as R&D and innovation (BarcenaRuiz and Espinosa, 1996). Moreover, as a key fiduciary, the performance of executives is closely linked to their positions and salaries, making them susceptible to managerial myopia and short-term behavior, and thus actively avoiding innovation activities that are of long-term interest to the firm.

On how to promote enterprise innovation, the academic community has analyzed working capital management (Ju et al., 2013), corporate political association (Liu et al., 2021), industrial policy (Feng, 2019), option incentive (Wang et al., 2017), ownership structure (Lee and Gereffi, 2021), operating leverage (Zhu et al., 2021), stakeholders’ “innovation concerns” (Pan and Yang, 2021), and other potentially influencing factors. These factors include endogenous institutional elements such as corporate control activities in addition to exogenous shocks such as national and industrial policies (Cui et al., 2021; Francis et al., 2021). However, artificial intelligence, big data, cloud computing, and other digital technology applications have gradually attracted close attention worldwide in the past decade. These new concepts are representative technologies of the new generation of the technological, industrial revolution, shaping the era of the digital economy.

As a new trend, the digital economy will promote “digital industrialization” in the process of promoting “industrial digitalization.” It can accelerate technological innovation by adjusting managerial behavior and strategic decisions, promoting and protecting technological research and development, and providing technological infrastructure. While technological innovation, which generally occurs in enterprises, will promote a new round of upgrading, optimization, and even reshaping of the whole industry. The two are highly synergistic. Therefore, it is of great practical significance to study the impact of the digital economy era on enterprise innovation behavior.

We chose CiteSpace software (Chen, 2004) as a literature data analysis tool to explore the impact of the digital economy era on corporate innovation behavior in the last 11 years from 2010 to 2020 through bibliometric methods and scientific knowledge mapping methods. With the current research status and progress as the core goal, we are committed to answering the following questions: (1) How has the number of publications and frequency of citations on the impact of the digital economy era on firms’ innovation behavior changed between 2010 and 2020? (2) What are the main research directions in this field? (3) What are the key literature nodes in the impact of the digital economy era on enterprise innovation behavior? (4) How are the research hotspots changing and evolving?

## 2. The origin, concept, and influence of digital economy

The term “digital economy” can be traced back to the 1990s, when scholars initially considered it as a new economic model under the information technology revolution. Since 2010, the digital economy has gradually entered public awareness as an emerging concept in China. Since 2015, the frequency of the term has been growing exponentially. Digital industrialization and industry digitalization embody the essence of “innovation-driven.” At present, the country’s economic growth is slowing down and economic restructuring is urgently needed. In addition to labor, capital, and land, big data has gradually become a new factor of production. The report to the 19th National Congress of the Communist Party of China (CPC) officially called for building a “digital China” by deeply integrating the Internet, big data, artificial intelligence, and the real economy.

The digital economy is a new development mode driven by data as the core element. It creates new value and explores new rules through analyzing and mining data elements (Pang and Zhu, 2013). In the national policy discourse, the digital economy is interpreted as “the deep integration of real enterprises and mobile Internet, Internet of Things, big data, cloud computing and artificial intelligence,” which contains the characteristics of industrial integration, innovation drive and new economic form (He and Liu, 2019).

The economic impact of the digital economy needs to be explored in two aspects.

(1) Macroeconomic. First, the digital economy helps to tap the long-tail demand outside the traditional market and the needs of customers to promote industrial specialization, product customization, and multi-field collaboration (Cao, 2018; He and Liu, 2019; Zhang et al., 2019). Second, the digital infrastructure improvement will optimize the atmosphere of social and technological innovation and improve the enthusiasm and creativity of the society to participate in innovation and integrate into technological change (Yang, 2019; Jiao and Liu, 2020). Since 2014, the construction of the digital economy has leaped from the initial science and technology policies to industrial and innovation policies.

(2) Corporate governance. There have been relatively sufficient studies on how digital development adjusts managerial motivation and behavior, as well as macro and micro resource allocation. First, digital technology can effectively reduce the cost of entity operation and improve the operational efficiency of organizations by removing the cost of intermediary and trust (Qi et al., 2021). Second, the digital economy can help enterprises enhance information sharing and openness, reduce information search and collection costs, explore structured and unstructured information, and strengthen the collaborative and integrated management of “pre-event, in-event, and post-event.” Thus, the first type of agency cost and information asymmetry widely existing in traditional corporate governance can be effectively alleviated (Li, 2019). In recent years, through the trend of deep integration of digital economy and traditional business, scholars have found that digital technology “empowerment” can make the organizational structure and business operation model more optimized (flattening, openness, cross-border cooperation), and help to develop a new business model (Liu et al., 2019).

Previous studies have demonstrated how the digital economy can effectively empower enterprise innovation, involving enterprise operation cost control, technological efficiency improvement, organizational management enhancement, and business model optimization (Bharadwaj et al., 2013; Hess et al., 2016; Nambisan et al., 2017; Economics, 2019; Bertani et al., 2021; Matarazzo et al., 2021). We use the method of knowledge-mapping to quantitatively analyze the research results, hotspot migration, and future direction of the relationship between the two to provide practical guidance for the academic community to master the frontier of the field, and managers to adapt to the development of the digital era and take the path of independent innovation.

## 3. Research on enterprise innovation

The term “innovation” originally originated from Schumpeter’s “Theory of Economic Development” in 1912, while enterprise innovation has been a hot topic in academia in the past decade, including but not limited to product and technological innovation. The research on enterprise innovation is mainly divided into enterprise innovation motivation, influencing factors, and economic consequences.

Regarding the motivation of enterprise innovation, we mainly start from the psychological demand of innovation subject, economic motivation, and the market economy system.

(A) In the psychological needs of innovation subjects, Ou et al. (2016) argued that enterprise innovation is to realize the technology cluster effect to obtain relying resources in the enterprise innovation ecosystem. Zhang et al. (2019) concluded that whether innovation stems from the magnitude of net benefits of corporate green innovation activities versus strict government environmental regulations. Liu et al. (2020) concluded that employee innovation self-efficacy, job engagement, and organizational innovation climate configuration would affect employee innovation behavior. Duan and Zhuang (2021) found a correlation between short-term profit-seeking and innovation, such as managerial financial investment.

(B) Among economic motives, Dang et al. (2015) found that enterprise innovation tends to be complementary to firms seeking political connections, and that anti-corruption campaigns increase the cost of political and corporate interactions, creating a “push-back mechanism” for their innovation. Liu (2016) found that to obtain tax incentives and financial subsidies, especially strategic emerging listed companies have a higher motivation to make a series of innovative changes in order to obtain policy subsidies. Zheng and Li (2015) and Yang et al. (2017) also confirmed the driving force of government subsidies on enterprise innovation. Pan and Yang (2021) also have the purpose of responding to the concerns of enterprise stakeholders on enterprise innovation. Wu and Dong (2020) also found that enterprise innovation behavior is partly driven by the supervision of independent directors’ social networks.

(C) In the market economy system, Yi and Liu (2015) found that the financial system can promote the industrial transformation and upgrading of enterprises through “horizontal effect” and “structural effect.” Peng and Zhu (2021) argued that market competition puts pressure on latecomers to survive, and such enterprises need to build alliances to realize innovation and make up for technological disadvantages. Jin et al. (2021) also believed that environmental pressure on radical innovation enhances the chaos of innovation evolution. Shao et al. (2021) found that the dynamic change process between supply and demand would lead to “disruptive innovation” in enterprises.

As for the influencing factors of enterprise innovation, the academic circle has made achievements in external supervision, external cooperation, and internal financial management.

(A) In terms of financial management, Ju et al. (2013) suggested the significance of working capital management in alleviating financing constraints and thus promoting innovation capital. Francis et al. (2021) found that organizational capital adequacy had a positive impact on corporate innovation. Zhu et al. (2021) found the positive significance of financial liabilities on innovation starting from external economic leverage. Duan and Zhuang (2021) believed that short-term profit-seeking, such as managerial financial investment, was negatively correlated with innovation.

(B) In terms of political association and external supervision, Yuan et al. (2015) and Yang et al. (2017) found that government subsidies and tax incentives would increase the motivation for enterprises to innovate, and enterprises would take the initiative to innovate for policy benefits. The political association also provides a green channel for such activities. Li and Zheng (2016) showed that import competition motivates enterprises to make high-quality innovations, and the incentive effect of import competition is more significant for enterprises vulnerable to competition and with high total factor productivity. In addition, they argued that selective industrial policies only motivate firms to innovate strategically, and the increase in patent applications can improve enterprises’ market value, promote technological progress, and achieve substantial innovation of competitive advantage. Pan and Yang (2021) also confirmed that “innovation concerns” of internal and external stakeholders were also potential influencing factors.

For the economic consequences of innovation activities, Piva and Vivarelli (2017) proposed a good dynamic correlation between R&D investment and employee employment. Xu (2021) pointed out that in terms of innovation input, talent and capital investment had a significant contribution to per capita subsistence consumption and changed consumption demand. Li et al. (2021) believed that the development of innovative activities such as technology credit would further promote the growth of enterprises, stimulate organizational model changes, and gain government support. Shao et al. (2021) argued that disruptive innovation could promote enterprises’ discontinuous transformation and realize the reorganization and allocation of various production factors. Berman et al. (2021) believed that the necessity of innovation could promote enterprises to form technological cooperation alliances and industrial agglomeration effect. The innovation agglomeration effect would promote market structure change.

## 4. Data sources and methods

### 4.1. Data sources

We investigate the impact of the digital economy era on firms’ innovative behavior from 2010 to 2020. We selected SSCI and A&HCI, two major citation databases within the Web of Science (WoS), which are internationally recognized and reflect the level of scientific research, as our search sources.

After comparing the literature data obtained by various search methods, we identified the search terms TS = (“digital economy” and “innovation”). The document type was “article,” the time frame was “2010–2020,” and the language was “English.” Our run time was 2 November 2021. We manually removed irrelevant documents and materials including conference abstracts, letters, data papers, books, and news in order to ensure the integrity, representativeness, and academic nature of the data, and obtained 725 valid literature data. Using CiteSpace software to de-process the collected 725 pieces of literature, we found that there were no duplicates. We, therefore, received 725 valid papers. Moreover, we selected 2010–2020 as the time parameter in CiteSpace software, 1 as the Year Per Slice, and Pathfinder as the pruning method for the subsequent analysis.

### 4.2. Methods

First, we present the statistics of the number of annual publications and the frequency of citations of the research related to the impact of the digital economy era on enterprise innovation behavior in the last 11 years using the bibliometric method, and analyze the changing trend of the two literatures over time from 2010 to 2020. Our aim is to understand the development status and research process of the impact of the digital economy era on enterprise innovation behavior in the last 11 years.

Subsequently, in the scientific knowledge graph analysis, the CiteSpace 5.8.R3 network visualization tool was used to visualize word frequency statistics and co-occurrence networks for two node types: cited literature (clustering analysis of research directions and key node literature analysis) and keywords (analysis of research hotspot evolution), respectively. In our visualization scheme, we use the node size to represent the word frequency (for both types of nodes represent the number of citations and the frequency of keyword occurrences, respectively). In addition to word frequency, centrality is also one of the indicators reflecting the importance of nodes in the network. In the CiteSpace visualization scheme, this indicator strictly refers to betweenness centrality, which quantifies the degree to which a node falls on the shortest path between any nodes in the network. In the cluster analysis, we also discuss two structural indicators of the clustering network, Q and Mean Silhouette. Among them, the former reflects the significance degree of each cluster formed in the network, and the latter reflects the homogeneity degree of nodes within the cluster, which together indicate the quality of the formed clusters (Chen et al., 2010).

## 5. The impact of the digital economy on enterprise innovation behavior: Results and analysis

### 5.1. Analysis of published quantity and cited frequency

**FIGURE 1:**
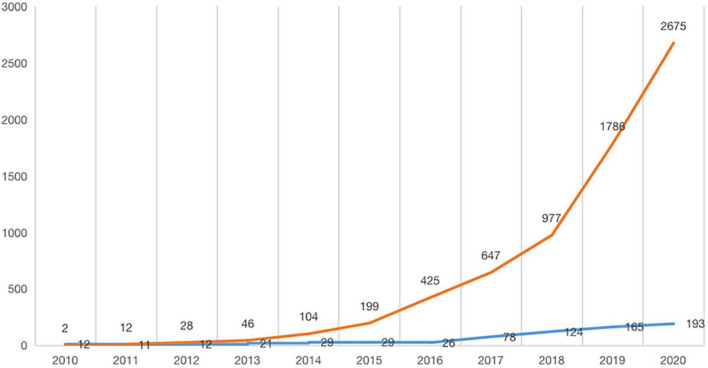
Annual statistics on the number of publications and frequency of citations.

We conducted annual statistics on the number of publications and citation frequency of 725 literature on the study of digital economy on enterprise innovation behavior to reveal the growth and aging pattern of the literature. And we make predictions on the future prospects and trends of the field based on the understanding of the development of the digital economy on enterprise innovative behavior to date. In Figure 1, citation times indicates the cumulative frequency of all relevant literature in that year.

Overall, the number of literature publications and the frequency of citations in the field from 2010 to 2020 showed an upward trend. The number of literature publications is in an overall increasing trend during the 11 years, especially since 2018, the research has been rapidly growing. Among them, the number of literature publications experienced significant growth in two time periods, 2015–2018 and 2018–2020, with an increase (rate) of 95 (327.59%) and 69 (55.65%), respectively. Therefore, we can divide the change in the digital economy on the amount of enterprise innovation behavior publication into three stages: the first growth period (2010–2015), the second growth period (2015–2018), and the third growth period (2018–2020). Until around 2015, as an emerging concept, the impact of the digital economy on corporate governance has not been widely recognized, and the research is scattered. With the application of the digital economy in many traditional industries, production efficiency is gradually optimized, and operating costs are reduced. Managers at the head of the industry begin to widely use digital technology to replace traditional, repetitive, and process-based manual operations, such as “Internet + “embedded in traditional banking, cloud computing impacts manual accounting bookkeeping. At the end of 2014, the academic circle began to pay close attention to the trend of “industry digitalization” and “enterprise digitalization transformation.” The number of relevant literature increased to 124, and the cited literature reached 977 by 2018. In the third growth period, the number of papers published on related topics in academia surged to 193 in 2020, which exceeded the number of publications in all years before 2017. In the rapid promotion and application of digital technology, “digital” is also regarded as a valuable resource to be tapped–big data, machine learning, artificial intelligence, and other fields represent cutting-edge algorithms centered on data resources.

The citation frequency of the literature has increased in a “J” shape, nearly 100 times in 11 years, with a total of 9,994 citations (excluding self-citations, which is 9,828) for 725 papers. In particular, citations increased by 1,698 in the most recent period from 2018 to 2020. This result shows that the literature on the two is attracting increasing attention and tends to heat up.

### 5.2. Main clusters of research directions

**FIGURE 2:**
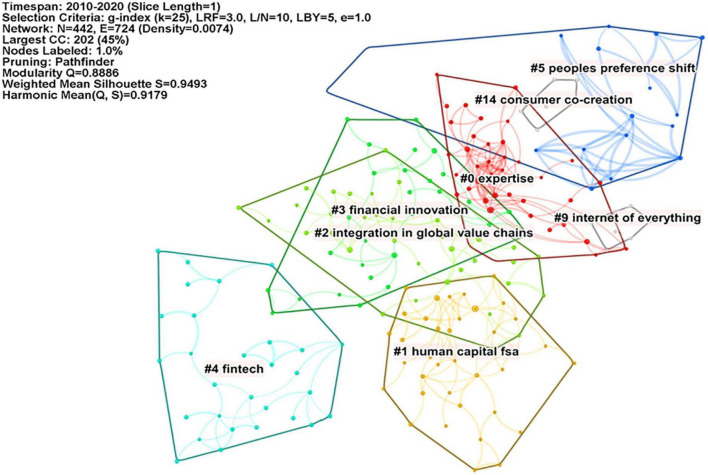
Statistics of literature co-citation network.

Our study used CiteSpace to conduct a co-citation analysis of sample literature. The period was from 2010 to 2020, with a 1-year time slice, a node type of “Reference.” The threshold was set as “references with top 50% citation times were selected from each time slice. To simplify the network and highlight the main features of the network, we use the “pathfinder” algorithm and the “pruning the merged network” strategy to prune the network, while other parameters are kept as default. After running, we obtained the co-citation network map of the digital economy on enterprise innovation behavior. Automatic clustering was carried out based on the network map, and the TF*IDF weighting algorithm was used to extract the clustering labels. Finally, the literature co-citation network clustering map was generated, as shown in Figure 2. The network modularity index *Q* = 0.8886 (>0.3) indicates that the clustering structure of the co-cited network map is very clear. The mean Silhouette indicator means Silhouette = 0.9493 (>0.5), which indicates good homogeneity within each cluster.

We only summarize the eight automatic clusters that contain nodes with a number greater than or equal to six in the graph. They represent the eight main research directions in the field from 2010 to 2020. Among them, the silhouette of each node is greater than 0.9. This result indicates that the index is very good in the homogeneity of these clusters. The number of nodes, silhouette index, label, and other information in each of the eight clusters is summarized in Table 1.

**TABLE 1:** Clustering of the eight main research directions.

Clustering	Number of nodes	Silhouette	Cluster identifier
0	38	0.941	Expertise
1	38	0.949	Human capital FSA
2	37	0.936	Integration in global value chains
3	29	0.911	Financial innovation
4	29	0.992	Fintech
5	17	0.957	People’s preference shift
9	8	1.000	Internet of everything
14	6	0.974	Consumer co-creation

The eight cluster identifiers are expertise, human capital FSA, integration in global value chains, financial innovation, fintech, people’s preference shift, internet of everything, and consumer co-creation. They can be subdivided into different subfields according to the research direction.

As the three clusters containing the largest number of nodes: integration in global value chains, expertise, and human capital FSA, reflect the emergence and promotion of the digital economy in the global field, affecting the division of labor and the integration and allocation of human resources in global value chains. However, the number of nodes in financial innovation and fintech are both 29, with a silhouette of 0.911 and 0.992, respectively. The result indicates that the research on digital finance has gone out of the “blue sea area” with abundant achievements, but its marginal innovation value may decrease. People’s preference shift, internet of everything, and consumer co-creation illustrate how digital technology can be used as a tool to improve the efficiency of an organizational operations. It can also change the profit model of enterprises, shape the new life model of the masses, and lead people to create a new round of high value-added.

### 5.3. Literature analysis of key nodes

In the literature co-citation network map (Figure 2), we extracted the literature with the top 10 citations. At the same time, these nodes also have high citation frequency. In CiteSpace, these key nodes with high centrality are defined as the pivotal point that controls the transition of the field from one period to another (Chen, 2004). In our study, these key nodes play the role of “bridges” –they are the bridges between literature with different research topics and form co-citation relationships with multiple literatures. They are also likely to be the bridge between the past and the future in digital economy research on enterprise innovation behavior. To some extent, these key nodes represent a hot topic for some time and control the development of the hot topics between the two periods.

Table 2 shows the top 10 key node literature cited. According to the research content, it can be broadly classified into three types: theoretical analysis of digital economy itself, exposition of the application of digital economy in enterprise innovation governance (belonging to the field of theoretical expansion), and judgment of digital economy serving social consumer groups (belonging to the practical tracking).

**TABLE 2:** Top 10 citations in research fields.

Ranking	Title	The first author	Journal source	Year	Times of citation
1	A neo-schumpeterian perspective of innovation, entrepreneurship and entrepreneurial marketing in the age of digitization	Parker, G. G	International Journal of Business Environment	2016	2
2	The sharing economy: Why people participate in collaborative consumption	Hamari, J	The Journal of the Association for Information Science and Technology	2016	1,133
3	The rise of the Platform Economy	Kenney, M	Issues of Science and Technology	2016	282
4	Multi-sided platforms	Hagiu, A	International Journal of Industrial Organization	2015	7
5	The sharing economy: A pathway to sustainability or a nightmarish form of neoliberal capitalism?	Martin, C. J	Ecological Economics	2016	547
6	Toward a theory of ecosystems	Jacobides, M. G	Strategic Management Journal	2018	396
7	Networks, platforms, and strategy: Emerging views and next steps	Mcintyre, D. P	Strategic Management Journal	2017	227
8	Sharing Economy: The End of Employment and the Rise of Crowd-Based Capitalism	Sundararajan, A	Sharing Economy: The End of Employment and The Rise of Crowd-Based Capitalism	2016	224
9	Putting the sharing economy into perspective	Frenken, K	Environmental Innovation and Societal Transitions	2017	11
10	You are what you can access: Sharing and collaborative consumption online	Belk, R	Journal of Business Research	2014	1,171

From the above analysis, it can be found that the literature content of these key nodes is mostly the basic theory of digital economy and the expansion and integration of the basic theory, which precisely reflects the role of these nodes with high school mentality as the “bridge” connecting different periods and different research topics. In these literatures with a high school mentality, the research objects are mostly in-group and out-group based on specific social situations (to a large extent, this is to ensure the internal validity of the research to clarify the basic causal relationship). However, in the literature on enterprise innovation behavior of the digital economy in the nearly 11 years from 2010 to 2020, more studies combining specific social issues began to emerge, such as the change of profit model, the emergence of sharing economy, the integration of multi-agent new economic ecology, virtual capital operation, etc. From the theoretical laboratory context, researchers focus on the digital economy’s innovation behavior on enterprises among specific social groups (such as consumers, creditors, investors, suppliers and marketers, governments, and other stakeholders). These key nodes have laid a solid theoretical and methodological foundation for the growing digital economy literature on enterprise innovation behavior in the following 20 years, especially for those who verify the basic theoretical results in specific scenario issues. Later, the themes of these studies gradually became the research hot in the field of enterprise innovation behavior in the digital economy. The shared and flat governance concept alleviated the information asymmetry between enterprises and society, impacted the traditional vertical framework management, and strengthened the cognition of “autonomy” and “initiative” among ordinary employees of enterprises. It provides a certain degree of guidance for the direction of follow-up research.

### 5.4. Evolution analysis of hotspots

Research hotspots refer to the research topics closely related and concerned by a large number of literature in a certain period. The analysis of research hotspots is helpful for researchers in this field to grasp which topics have received a great deal of attention from other scholars at what time and how different research perspectives in this field develop and change.

**FIGURE 3:**
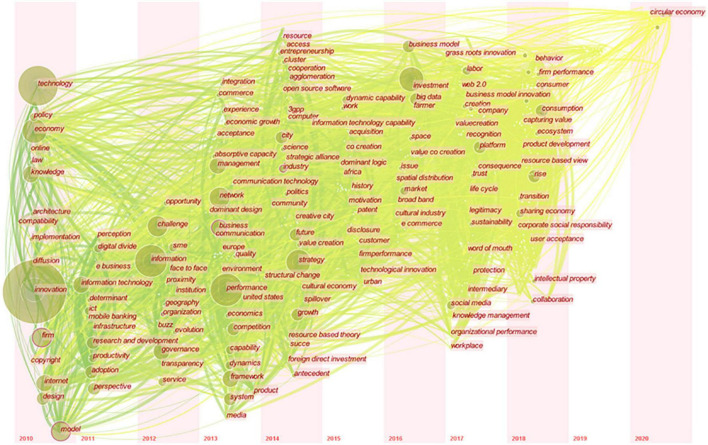
Time-zone diagram of keyword co-occurrence network.

In our study, we determine the research hotspots in the field of digital economy’s impact on enterprise innovation behavior by analyzing the word frequency of keywords. These keywords are often the epitome of the research topic and the high generalization of literature content. CiteSpace software was used, the period was set as 2010–2020 (Slice Length = 1), node type was selected as “Keyword,” and the threshold was set as “keywords with the top 50% frequency in each time Slice.” In the network diagram, the Pathfinder function in the connection was Pruning. To observe the evolution of research hotspots in the time distribution, we selected the “TimeZone” view to draw the TimeZone diagram of the keyword co-occurrence network, as shown in Figure 3.

It can be found in Figure 3 that keywords with high frequency (large node size) are mainly concentrated in the time zone from 2010 to 2014. The number of new keywords in the literature increased significantly in 2013. Among them, the keywords innovation, technology, economy, firm, model, strategy, and knowledge appeared more than 30 times for the first time in 2010. Moreover, the keyword nodes in the time zone in 2010 are closely connected with the keyword nodes in the time zone in the following 10 years. This result indicates that the topics of the 2010 and previous studies are likely to be relatively basic and broad, paving the way for subsequent studies. The theme and perspective of the follow-up research are largely related to the research topics in 2010 and before, which may be an extension of it or a reflection on practical problems.

Table 3 summarizes the high-frequency keywords of the digital economy on enterprise innovation behavior for the first time in the literature from 2010 to 2020. Accordingly, in chronological order, we can analyze the hot spots in each stage of the research on the digital economy on enterprise innovation behavior.

**TABLE 3:** High-frequency keywords first appeared each year (frequency >3).

Year	High-frequency keywords (frequency)
2010	Innovation (184), Technology (64), Economy (43), Firm (37), Model (33), Knowledge (31), Internet (21), Design (20), Policy (17), Diffusion (11), Law (8), Implementation (6), Online (5), Architecture (4)
2011	Information technology (26), Adoption (22), Ict (20), Perspective (15), Determinant (14), Research and development (11), Productivity (9), Digital divide (8)
2012	Information (34), Challenge (24), Governance (15), Organization (11), Geography (8), Service (8), Evolution (5), Opportunity (4)
2013	System (31), Network (29), Competition (20), Business (18), Dynamics (12), Capability (12), Product (8), Economics (6), Absorptive capacity (6), Economic growth (6), Acceptance (6), Commerce (5)
2014	Performance (33), Future (20), City (19), Entrepreneurship (10), Cluster (8), Science (7), Community (5), Antecedent (5)
2015	Strategy (33), Management (29), Industry (12), Dynamic capability (7), Customer (4)
2016	Impact (36), Business model (19), Big data (18), E commerce (3)
2017	Framework (23), SME (15), Growth (13), Market (10), Sustainability (8), Labor (6)
2018	Sharing economy (15), Consumption (11), Rise (10), Social media (8), Uber (7), R&D (6), Transformation (6), Work (5), Experience (5), Space (5), Un captured GDP (5), Value creation (5), Behavior (4), Intermediary (4), Integration (4), Consumer (4), Product development (4)
2019	Platform (11), Politics (8), Creation (6), Transition (6), Inclusion (4), Investment (4)
2020	Ecosystem (5), Power (4)

In general, we can divide the evolution of research hotspots of the digital economy on enterprises’ innovation behavior into three periods according to the time when keywords first appear: (1) Enterprises’ perception of the digital economy; (2) Enterprises’ familiarity with and application of digital technology; (3) Enterprises realize the digital transformation stage.

### 5.5. How the digital economy influences enterprise innovation behavior

Our study examines the “digital economy” and “innovation,” and our findings indicate that the digital economy can significantly improve enterprise innovation. It has been demonstrated that the digital economy accelerates information exchange and thus facilitates the spillover of knowledge. Additionally, enterprises can improve innovation efficiency by improving their capabilities in learning knowledge and experience.

(1) As digital technology develops, a convenient platform for information exchange is provided. In addition to reducing the cost of searching for external information, digital technology promotes the agglomeration and diffusion of resources and technologies, therefore enhancing enterprise innovation efficiency.

(2) As a result of its Internet platform, the digital economy promotes two-way communication between the supply and demand of products and stimulates consumers’ demand for product diversification. It is essential to enable enterprises to change product solutions according to consumer needs in a timely manner to optimize business opportunities and improve research and development efficiency.

(3) With the development of cutting-edge technologies such as the Internet, big data, cloud computing, and artificial intelligence, the digital economy is able to encourage enterprises to utilize digital intelligent equipment in their products more effectively. Furthermore, it may also be used as a way to optimize the matching of information on the market, thereby enhancing the innovation capabilities of enterprises.

(4) Due to its social interaction and information channel effects, the digital economy accelerates the circulation and dissemination of data elements and provides a basis for enterprise innovation decisions. Moreover, it eliminates barriers to innovation and collaboration. As a result, the region may be able to promote the innovation and entrepreneurship activities of firms in the area. There may be a demonstration effect throughout the surrounding area, and the spillover dividends associated with innovation may also manifest.

(5) As social networks positively influence enterprise innovation, the development of a digital economy may provide enterprises with a higher level of digital access, effectively enhance the connectivity of innovation networks, and facilitate the attraction of partners by providing a digital economy. Consequently, external communication structures will be accelerated, external information and resources will be attracted, and the innovation potential of the digital economy will be maximized.

## 6. Development trend of enterprise innovation behavior research in the digital economy era

### 6.1. Discussion and conclusion

As the digital economy has grown rapidly and has been incorporated into products and services, the academic community is increasingly concerned about the impact of digital economy on enterprise innovation. We investigated the number of publications and citations relevant to the impact of the digital economy on enterprise innovation behavior from 2010 to 2020, the main research directions, the key node literature, and the evolution of research hotspots. According to the above results, the main conclusions are as follows.

First, from the overall overview of literature published and cited, the research heat in the field is growing rapidly. From 2010 to 2020, the number of publications is generally on the rise. In particular, the number of citations in the literature has increased rapidly by nearly 100 times in 11 years, indicating that literature in the field has attracted more and more attention from scholars. The digital economy has its potential for value mining in promoting digital transformation of enterprises and industries. Digital industrialization has good research prospects and marginal contribution.

Second, through the literature co-citation analysis, we find that the research on enterprise innovation behavior in the digital economy has formed a relatively complete basic theory and research framework. Cluster analysis shows that the research is distributed in several basic research directions, including global human resource allocation, fintech application, social group behavior changes, sustainable economic model rise. There is considerable overlap among all directions, and most of the specific literature nodes are extensions of existing research directions. Further analysis of key node literature shows that most of the key node literature was published during 2014–2018, mainly including the basic theory and the extension of the basic theory of digitalization, and the interpretation of specific application scenarios of digitalization. These key literature nodes are the “bridges” connecting the past and the future, and they control the transition from one period to another in the development path of the digital economy to the theory of firm innovation behavior.

Third, the analysis of the time zone atlas of keyword co-occurrence network and the high-frequency keywords in each time zone shows the evolution of research hotspots from theory to practice, from the basic model to interdisciplinary and interdisciplinary attempts. Research hotspots are increasingly able to reflect the real life of human beings and social issues. The cross-disciplinary attempts have led to the increasing ability of the digital economy to explain the theory of corporate innovative behavior in different contexts to real problems. For example, structural changes in the financial industry, consumer preferences, corporate market competition, organizational operating models, and managerial behavior, etc. This development trend reflects the high degree of integration between emerging digital technologies and humanities and social sciences. Contemporary society is in a period of rapid development. Digital technology will impact traditional enterprise governance, and give impetus to innovation in areas such as upgrading corporate technology and optimizing management structures. It also put forward higher requirements. In the future, the research should be committed to revealing social phenomena through basic research and deepening the understanding of the impact process of the digital economy on enterprise innovation behavior, and exploring evidence-based promotion measures, and providing reasonable suggestions for boosting the “empowerment” enterprise innovation of digital economy.

It is evident from the review of the past 11 years that the research of digital economy has made certain achievements both in theory and in practice in terms of its impact on enterprise innovation behavior. Our study sorted out and enriched the understanding and analysis perspective of the development of digital economy on the micro subject of enterprises. However, research in this field still has great potential and development space in the long run. Future can be carried out from the following perspectives:

First, attention should be paid to ecosystems. The research can be combined with ecosystem analysis. In the above analysis, the term “ecosystem” first appeared in 2020. The enterprise is only a subsystem of the ecosystem, and its innovation activities need to be guaranteed by the normal operation of the ecosystem. In the early stage of the development of the digital economy, due to the small conflict between enterprises and the outside world and the weak ability of enterprises to transform the world, the ecosystem-related problems caused by the digital economy were not taken seriously. However, with the development of the digital economy, the impact of enterprise innovation on the ecosystem will become more and more significant. Future research should consider the embeddedness of the digital economy and attach importance to the mutual influence and restriction of the digital economy on enterprise innovation behavior, to achieve a relatively stable dynamic equilibrium state.

Second, attention should be paid to dynamic processes. Enterprise innovation activity is a dynamic process. Many problems of the digital economy on enterprise innovation behavior, such as the emergence and development of the digital economy and the relationship between the digital economy and enterprise innovation, involve time factors, such as lag effect, cross effect, proximal, or distal results, etc. Therefore, the research of digital economy on enterprise innovation behavior can use a computer, artificial intelligence and other technologies to capture the vertical, rich, constantly changing non-linear digital economy on enterprise innovation behavior process. Future research should pay attention to the complex development process of the digital economy on enterprise innovation behavior. Bringing the time effect into the longitudinal tracking research is conducive to more effectively exploring the causal relationship between variables.

Third, attention should be paid to different innovation decisions. More and more scholars focus on the environmental benefits, openness, and foresight of enterprises’ innovation activities by incorporating the frontiers of innovation into the study of enterprise innovation from the perspective of the digital economy. In recent years, there is a growing literature on green innovation, open innovation, destructive innovation, and breakthrough innovation. Therefore, we can consider further exploring the impact of digital economy on different innovation decisions of enterprises such as green innovation, open innovation, disruptive innovation, and breakthrough innovation to provide empirical support for subsequent research.

Fourth, heterogeneity should be paid attention to in the future. With the in-depth promotion of the digital economy on enterprise innovation behavior, the rapidly growing market demand will force the promotion of enterprise innovation practice research, and the research results will better connect with the regional practice. The research on the practical application of digital economy in different countries/regions, different enterprise nature, and different economic system environments on the development ideas, existing problems, promotion strategies, and promotion paths of enterprise innovation behavior will also be gradually deepened. At the same time, the horizontal or vertical comparative research on enterprise innovation behavior by digital economy in different countries/regions, different enterprise nature, and different economic system environment will also be concerned. The cross-fertilization between theory and practice will contribute to the high-quality and in-depth development of the digital economy and enterprise innovation.

### 6.2. Research trend based on the Chinese context

Based on Chinese current national conditions, it is appropriate to test the significance of the digital economy on enterprise innovation behavior.

(1) China, as the second largest economy in the world, has surpassed the United States in terms of total GDP in purchasing power parity since 2015. Under the background of a slowing growth rate and pending adjustment of economic structure, micro-individual innovation is a necessary choice to promote economic transformation.

(2) At present, China has entered the rapid growth stage of the market economy system. There are still gaps between China and European and American capitalist countries in the capital market, infrastructure, and management, which requires policy optimization in aspects including but not limited to the digital economy.

(3) Since China’s reform and opening up, the system construction has always embodied the “fully mobilizing the factors of labor production,” “more work, more pay,” and “increasing the enthusiasm and creativity of workers.” As an exogenous impact, the digital economy can provide the sustainable driving force for enterprise operation and innovation from positive incentives and reverse forces.

(4) The Chinese system actively distinguishes between state-owned and non-state-owned property rights systems, and the state-owned economy plays a dominant role. There may be a difference in the marginal effect of digital economy development on the innovation behavior of these enterprises, which are yet to be tested empirically.

(5) At the end of 2020, the number of listed companies in Chinese Shanghai and Shenzhen stock Markets has reached 4,100, widely distributed. Different enterprises are affected by different geographical locations, provincial systems, regional economies. And there are also differences in executives’ characteristics, which can moderate the effect of digital development.

## 7. Conclusion and prospects

Enterprise innovation has been a hot topic in academia in recent years, and it is the core element of enterprise dynamic growth and economic growth. In the past 5 years, there has been literature focusing on the significance of managerial characteristics and behavior to enterprises’ innovation and development (Chesney et al., 2020; Gao et al., 2021), but these are mainly limited to endogenous self-selection behavior.

Our study investigates the relationship between “digital economy and enterprise innovation behavior,” and illustrates the promotion effect of digital construction on R&D and innovation activities. With the development of digital economy, enterprises and their stakeholders pay more and more attention to digital economy. It is an inevitable trend that digital economy will be widely used in enterprise innovation behavior in the future. It is helpful to consider the soft science factors such as executive motivation and external incentives in organizational management to study enterprises’ innovation production changes from the perspective of managerial behavior changes, which is instructive for enterprises’ strategic choices.

Meanwhile, if advanced technologies can be successfully applied to Chinese enterprises, research, development, and innovation will be required in conjunction with China’s national conditions, market conditions, and enterprise development. In the China 2025 Industrial Plan and the innovation practice of the 19th National Congress of the Communist Party of China, we call for the establishment of a digital construction system to better enhance the enthusiasm of enterprises to participate in innovation activities, to improve the social innovation atmosphere, to strengthen the driving force of innovation, and to realize the promotion of the strategy of “innovation power.”

## Data availability statement

The original contributions presented in this study are included in the article/supplementary material, further inquiries can be directed to the corresponding author.

## Author contributions

WY: research design, data collection, sorting, analysis, drafting the manuscript, supervision, and providing project funding. LZ: modifying the manuscript, suggestion, and supervision. CY: research idea, data collection, drafting, and modifying the manuscript. All authors contributed to the article and approved the submitted version.
